# Ageism and Undergraduate Attitudes towards Older Adults during COVID-19

**DOI:** 10.1093/geroni/igab046.2295

**Published:** 2021-12-17

**Authors:** Adam Shea, Aiping Yu, Jessica Strong

**Affiliations:** 1 University of Prince Edward Island, University of Prince Edward Island, Prince Edward Island, Canada; 2 University of Prince Edward Island, Charlottetown, Prince Edward Island, Canada

## Abstract

The COVID-19 pandemic has shed light on the far reaches of ageism in our society. The current study sought to better understand ageist beliefs in Canadian undergraduate students during the pandemic. As part of a larger survey on ageism, we conducted a thematic analysis on open-ended responses to the following questions: 1) “Has your relationship with older adults in your life changed as a result of the COVID-19 pandemic?” and 2) “Have you noticed that attitudes or opinions towards older adults in your community have changed as a result of the COVID-19 pandemic?” Students felt that older adults should be treated differently during the pandemic because they are seen as “high risk” or “vulnerable.” Furthermore, students felt that they needed to be more cautious around older adults because older adults need to be taken care of. Students expressed fear about transmitting the virus to older adults in their lives so chose to isolate from grandparents or avoid older adults in the community in an effort to keep them safe. Finally, examples of negative and positive ageism were present in responses. Negative ageism was seen in comments about how older adults were going to die anyway, the assumption that older adults need more help, and the belief that older adults should be staying home during the pandemic. Positive ageism was present where students realized the importance of their relationships with the older adults in their lives. Results are discussed using the framework of implicit and explicit ageism.

